# Lamotrigine-associated SJS/TEN overlap during valproate co-therapy managed with rhTNFR-Fc and therapeutic plasma exchange: A case report

**DOI:** 10.1097/MD.0000000000051050

**Published:** 2026-10-09

**Authors:** Fang Chen, Xiao Li

**Affiliations:** a Department of Dermatovenereology, Leshan Hospital of Traditional Chinese Medicine, Affiliated to Chengdu University of Chinese Medicine, Leshan, Sichuan, People’s Republic of China; b Intensive Care Unit, Leshan Hospital of Traditional Chinese Medicine, Affiliated to Chengdu University of Chinese Medicine, Leshan, Sichuan, People’s Republic of China.

**Keywords:** lamotrigine, severe cutaneous adverse reaction, SJS/TEN overlap, therapeutic plasma exchange, TNF-alpha inhibitor

## Abstract

**Rationale:**

Stevens-Johnson syndrome/toxic epidermal necrolysis (SJS/TEN) is a rare, life-threatening severe cutaneous adverse reaction. Evidence for escalation therapy in relapsing disease remains limited.

**Patient concerns:**

A 49-year-old man developed fever and a rapidly progressive generalized erythematous maculopapular eruption after lamotrigine was initiated directly at 50 mg once daily during concomitant valproate therapy.

**Diagnoses:**

At admission on August 3, 2023, no epidermal detachment was observed and SJS/TEN was not clinically recognized. On August 9 (hospital day 7), epidermal fragility, small blisters, and a positive Nikolsky sign led to recognition of the SJS/TEN spectrum, with detachment below 10%. Severity-of-illness score for toxic epidermal necrolysis was 1 at that time; age > 40 years was the only positive criterion. By day 12, detachment reached approximately 17% of the body surface area, supporting SJS/TEN overlap. The algorithm of drug causality for epidermal necrolysis score for lamotrigine was 5 (probable). Skin biopsy was declined.

**Interventions:**

During the initial diagnostic uncertainty on hospital days 1 and 2, lamotrigine was continued; it was discontinued on day 3 when a severe drug eruption was suspected. Intravenous immunoglobulin and intravenous methylprednisolone produced transient improvement. New epidermal detachment prompted methylprednisolone escalation and recombinant human tumor necrosis factor receptor-Fc fusion protein treatment. Relapse during corticosteroid tapering prompted 3 therapeutic plasma exchange sessions while corticosteroid therapy continued.

**Outcomes:**

Three therapeutic plasma exchange sessions were subsequently administered with continued corticosteroid therapy, after which the eruption cleared. The patient was discharged on hospital day 33 and had no relapse or reported sequelae at 3-month follow-up.

**Lessons:**

This case illustrates the evolution from a severe drug eruption to SJS/TEN overlap, reinforces the importance of reduced lamotrigine starting doses and slow titration during valproate co-therapy, and provides a clinical example of stepwise multidisciplinary rescue management in relapsing disease.

## 1. Introduction

Stevens-Johnson syndrome/toxic epidermal necrolysis (SJS/TEN) is characterized by epidermal necrosis with mucosal involvement and can be life-threatening.^[1]^ Early withdrawal of the culprit drug is essential, but systemic therapy remains debated because high-quality trial evidence is limited.^[2]^ Corticosteroids and intravenous immunoglobulin are commonly used.^[3]^ Tumor necrosis factor-alpha blockade and therapeutic plasma exchange (TPE) have been reported in selected severe or refractory cases.^[4,5]^ We describe a lamotrigine-associated case that required stepwise escalation, including TPE. This manuscript was prepared and revised in accordance with the Case Report checklist.

## 2. Case presentation

A 49-year-old man (65 kg) had hypertension, diabetes, and a cerebral infarction 9 months earlier with residual left hemiparesis. He had been taking aspirin and atorvastatin long term. Secondary epilepsy had been treated with valproate extended release 0.5 g twice daily for 5 months, but seizures persisted 2 to 3 times per month. Lamotrigine was, therefore, initiated directly at 50 mg once daily 1 month before admission. He denied drug or food allergy and had never smoked.

On lamotrigine day 31, approximately 1 day before admission, fever (38°C) and erythematous macules developed on the head, face, and neck. Within 24 hours, the eruption progressed to generalized erythematous macules and papules. On hospital day 1, cutaneous lesions involved approximately 90% of the body surface area (BSA), but no epidermal detachment was observed (Fig. 1). Mild conjunctival injection, submandibular lymphadenopathy, and pharyngeal congestion were present. Initial blood counts, biochemical tests, liver and kidney function, serum electrolytes, and arterial blood gases showed no clinically significant abnormalities.

**Figure 1.**
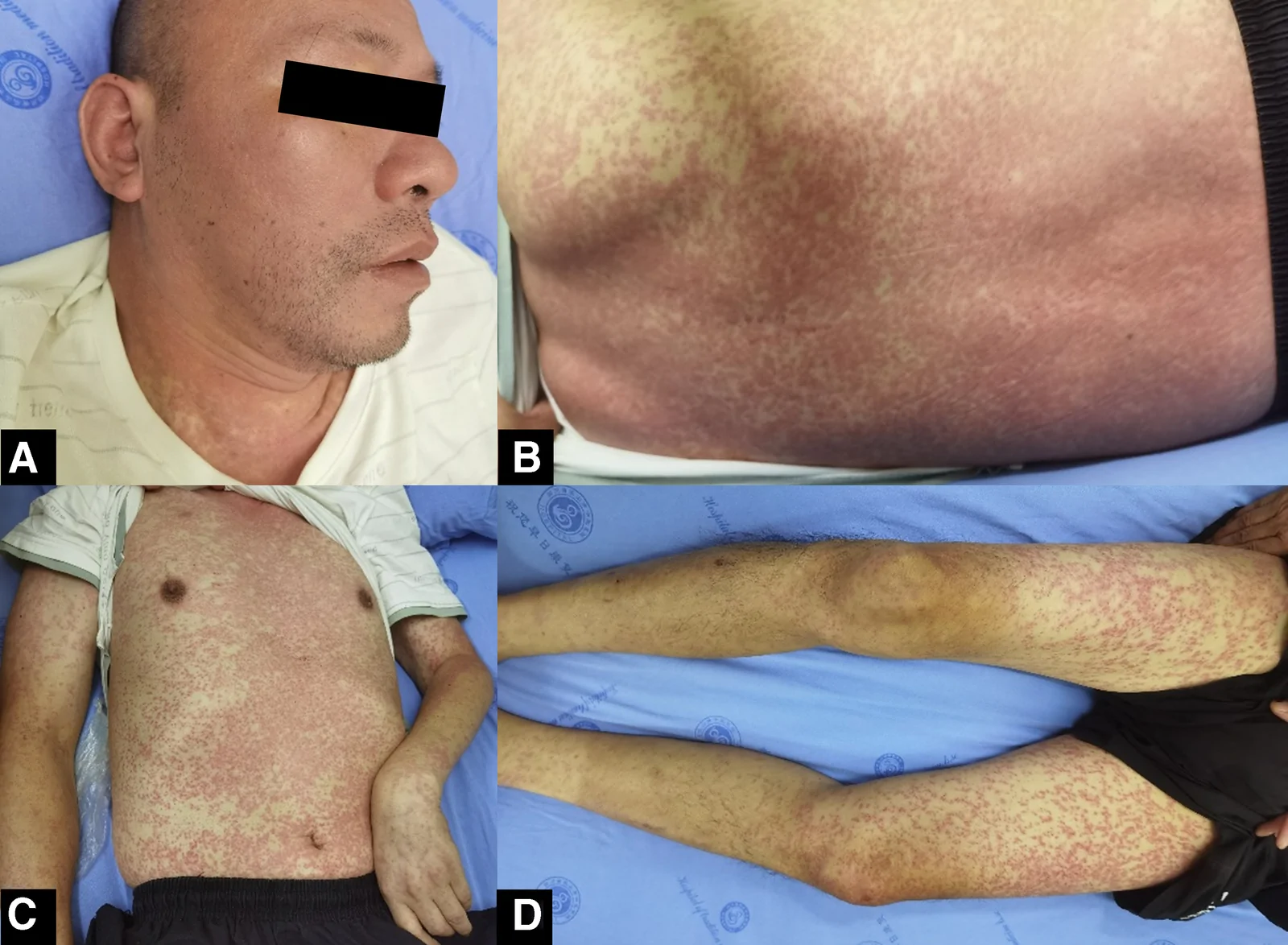
Clinical features on admission. Diffuse erythematous macules and papules over the head, face, and neck (A), with extensive cutaneous involvement of the trunk (B, C) and lower extremities (D).

During hospital days 1 and 2, the absence of epidermal detachment and limited mucosal findings initially suggested an infectious exanthem, and lamotrigine was continued. Available microbiological and serological findings did not support rubella, scarlet fever, or bloodstream infection. Supportive care comprised fluid and electrolyte management, wound care, antipyretic treatment, and pain control. As the eruption progressed, a severe drug eruption was suspected. Lamotrigine was discontinued on hospital day 3 and replaced with levetiracetam 250 mg twice daily after neurology consultation. From hospital days 3 to 5, intravenous immunoglobulin 0.4 g/kg/day and intravenous methylprednisolone 160 mg/day were administered before the SJS/TEN spectrum was clinically recognized. Fever resolved, and the eruption markedly regressed by hospital day 6 (Fig. 2).

**Figure 2.**
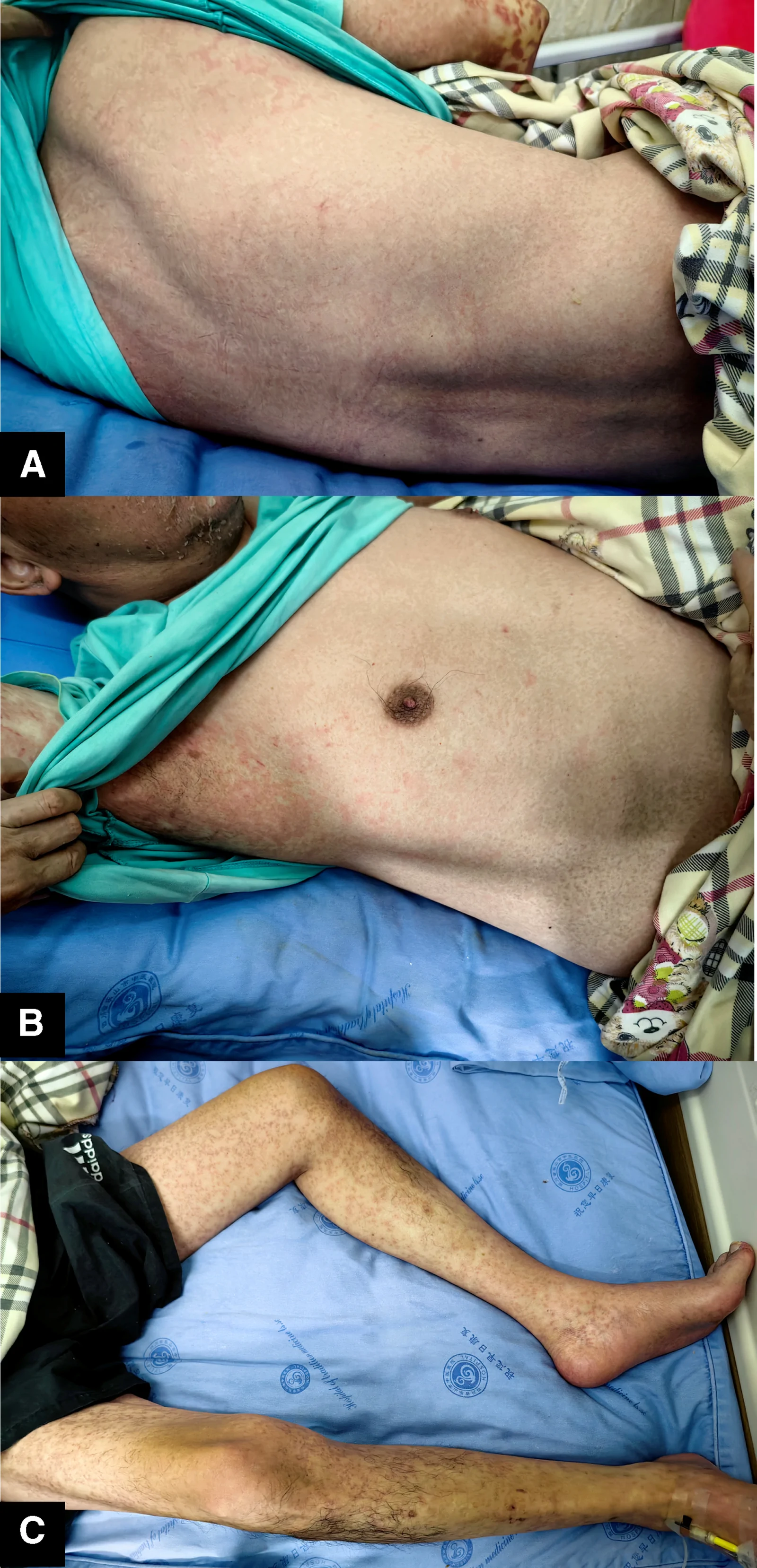
Improvement after initial systemic therapy. The eruption on the back (A) and chest/abdomen (B) had largely subsided, with marked improvement over the lower extremities (C).

On hospital day 7 (August 9, 2023), the eruption flared, with epidermal fragility and small blisters on the right ankle and left knee; the Nikolsky sign became positive. The SJS/TEN spectrum was clinically recognized at this time. The detached area was estimated at approximately 300 cm^2^ and remained below 10% of the total BSA. A severity-of-illness score for toxic epidermal necrolysis of 1 was calculated at that time, with age >40 years as the only positive criterion. The heart rate was 76 beats/min; serum urea, glucose, and bicarbonate were 6.21, 6.1, and 25.0 mmol/L, respectively. The patient had no malignancy. In response to progression, intravenous methylprednisolone was escalated to a maximum of 500 mg/day. Recombinant human tumor necrosis factor receptor type II-IgG Fc fusion protein (rhTNFR-Fc; Yisaipu, an etanercept biosimilar; 3SBio Inc., China) was initiated at 25 mg subcutaneously. It was administered on hospital days 7, 10, and 13 (August 9, August 12, and August 15, 2023), for a total dose of 75 mg. By hospital day 12, mild erosions involved the conjunctiva, oral mucosa, and genital area, with scattered epidermal detachment over the elbows, left hip, and lower legs. The detached area had reached approximately 17% of the BSA, supporting classification as SJS/TEN overlap (Fig. 3).

**Figure 3.**
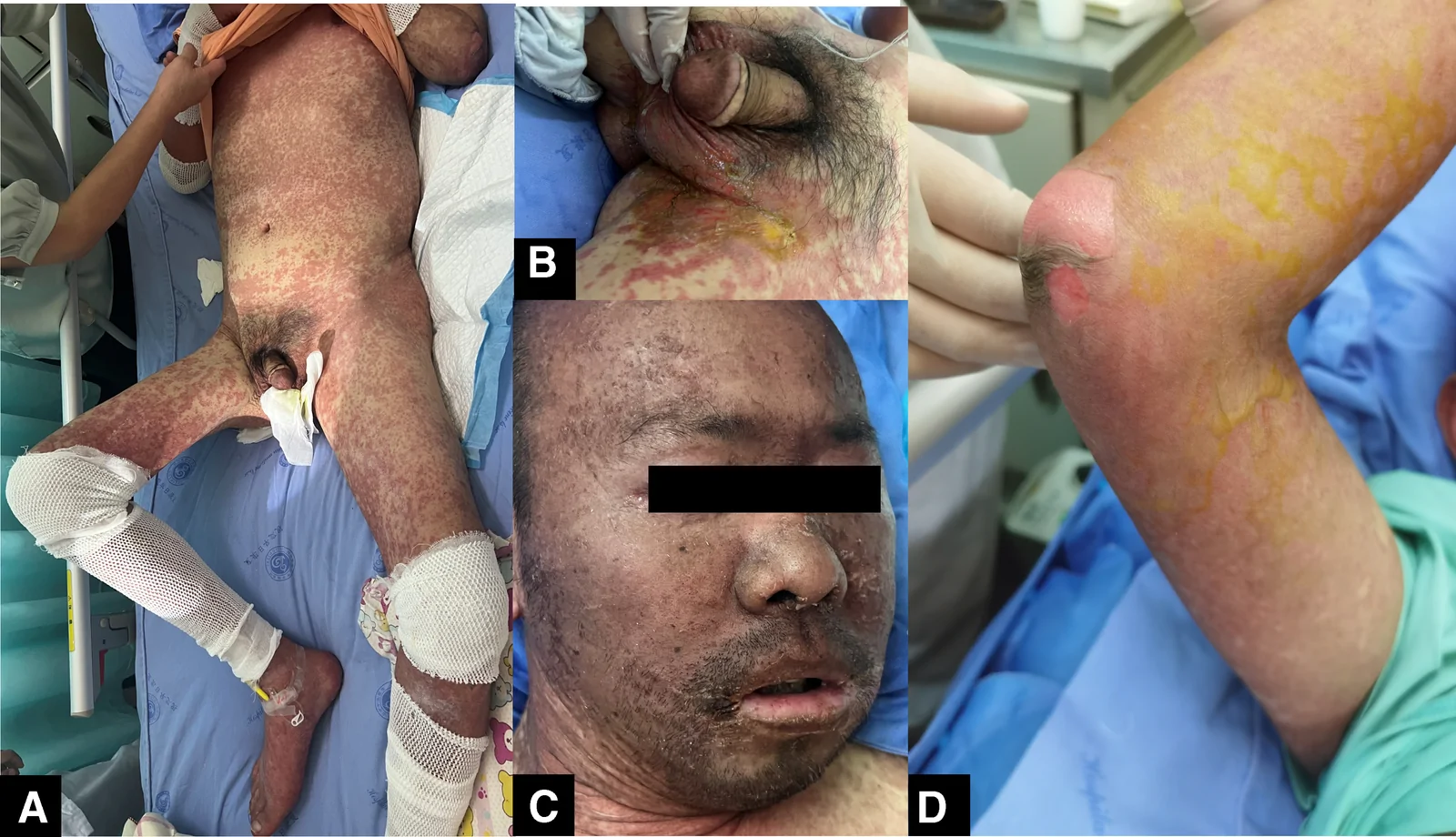
Progression with mucosal involvement and epidermal detachment. Widespread erythema over the trunk and lower limbs (A). Genital mucosal involvement with erosions and crusting (B). Facial involvement with perioral crusting (C). Localized epidermal detachment over the elbow (D).

Desquamation and reepithelialization occurred during hospital days 14 to 20, while intravenous methylprednisolone was tapered to 160 mg/day. On hospital day 21, erythematous lesions recurred during corticosteroid tapering. Oral methylprednisolone 32 mg/day was added while intravenous methylprednisolone was continued at 160 mg/day. On hospital days 24 to 26, 3 consecutive TPE sessions were performed in the intensive care unit while corticosteroid therapy continued. Each session used heparin anticoagulation and 2000 mL of fresh frozen plasma (approximately 30 mL/kg). Erythema subsequently cleared, with no procedure-related hypotension, bleeding, or allergic reactions. The daily methylprednisolone dose was then reduced to 120, 80, 80, and 40 mg on hospital days 26 to 29, respectively. The patient was discharged on hospital day 33 while receiving prednisone acetate 20 mg once daily (Fig. 4).

**Figure 4.**
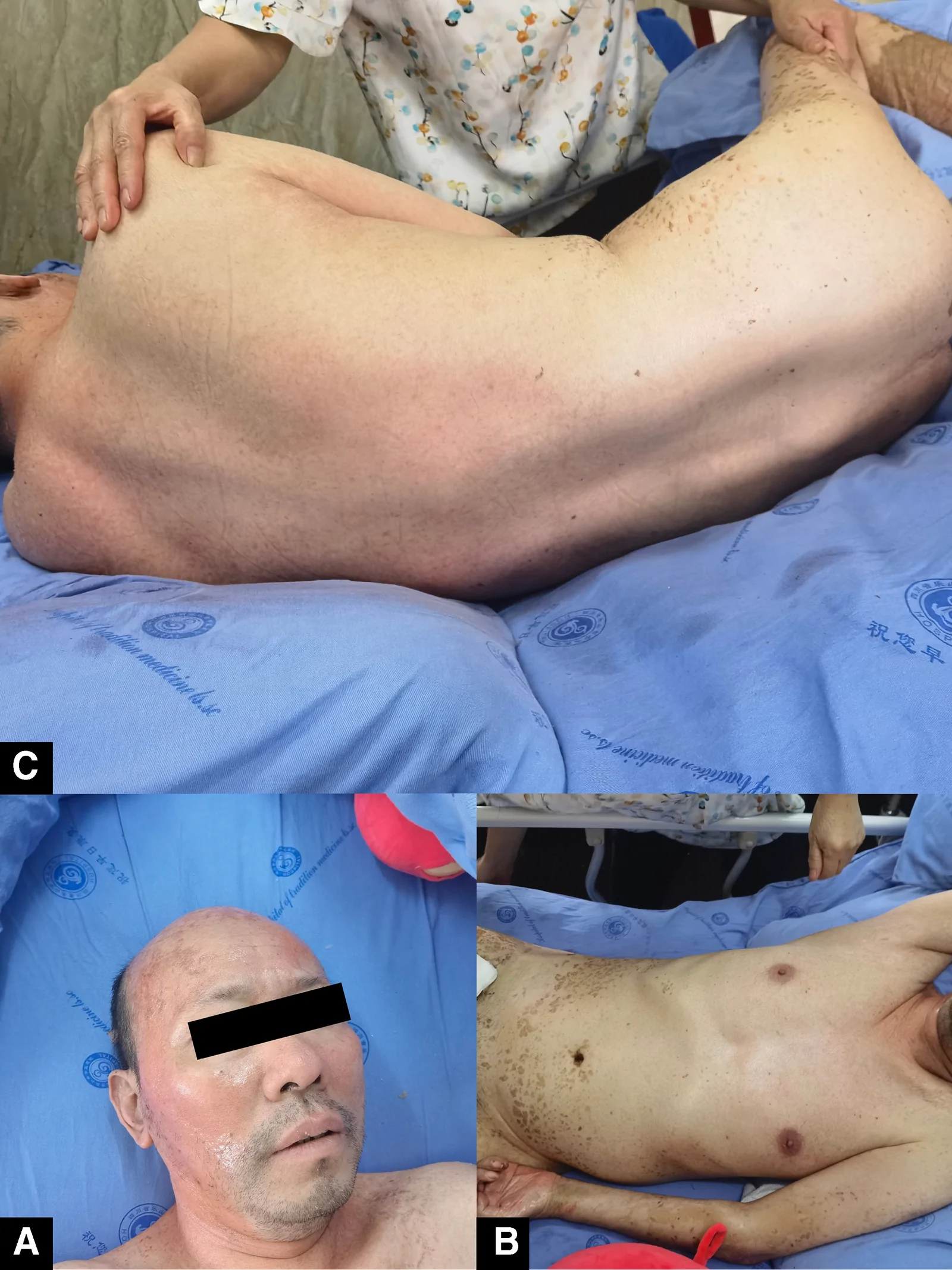
Outcome at discharge (day 33). Resolution of facial erythema (A) and clearance over the chest/abdomen (B) and back (C), with residual fine scaling.

At 3-month follow-up, no relapse or sequelae were reported. The complete clinical course is summarized in Table 1.

**Table 1:** Chronological clinical course, treatment escalation, and follow-up.

Time point	Clinical events, interventions, and outcomes
Before admission and symptom onset	Valproate XR 0.5 g twice daily was used for 5 months. Lamotrigine 50 mg once daily was started 1 month before admission. On lamotrigine day 31, fever and erythematous macules progressed within 24 hours to a generalized eruption.
Hospital days 1–3	At admission, erythema involved approximately 90% BSA, with no epidermal detachment observed. An infectious exanthem was initially considered, and lamotrigine was continued on days 1 and 2. Progression suggested a severe drug eruption; lamotrigine was discontinued on day 3 and replaced with levetiracetam.
Hospital days 3–6	Intravenous immunoglobulin 0.4 g/kg/day and intravenous methylprednisolone 160 mg/day were administered on days 3–5. Fever resolved, and the eruption markedly regressed by day 6.
Hospital days 7–13	On August 9 (day 7), epidermal fragility, blisters, and a positive Nikolsky sign prompted recognition of the SJS/TEN spectrum. Detachment was approximately 300 cm^2^ and remained below 10% BSA. SCORTEN was 1, with age >40 years as the only positive criterion. Intravenous methylprednisolone was escalated to a maximum of 500 mg/day, and rhTNFR-Fc 25 mg subcutaneously was administered on days 7, 10, and 13.
Hospital days 12–20	By day 12, mucosal erosions were present and epidermal detachment had reached approximately 17% BSA, supporting classification as SJS/TEN overlap. Desquamation and reepithelialization followed, and intravenous methylprednisolone was tapered to 160 mg/day.
Hospital day 21	Erythematous lesions recurred during corticosteroid tapering. Oral methylprednisolone 32 mg/day was added while intravenous methylprednisolone was continued at 160 mg/day.
Hospital days 24–29	Three consecutive TPE sessions were performed on days 24–26 while corticosteroid therapy continued (fresh frozen plasma, 2000 mL/session). Erythema subsequently cleared without a procedure-related adverse event. The daily methylprednisolone dose was reduced on days 26–29.
Hospital day 33 and follow-up	The patient was discharged while receiving prednisone acetate 20 mg/day. No relapse or sequelae were reported at 3-month follow-up.

BSA = body surface area, rhTNFR-Fc = recombinant human tumor necrosis factor receptor-Fc fusion protein, SCORTEN = severity-of-illness score for toxic epidermal necrolysis, SJS/TEN = Stevens-Johnson syndrome/toxic epidermal necrolysis, TPE = therapeutic plasma exchange, XR = extended release.

## 3. Discussion

Lamotrigine is a well-recognized trigger of severe cutaneous adverse reactions, including SJS/TEN.^[6]^ Valproate inhibits lamotrigine glucuronidation, reduces clearance, and prolongs elimination half-life, thereby increasing lamotrigine exposure.^[7]^ For adults receiving valproate, current prescribing information recommends lamotrigine 25 mg every other day during weeks 1 and 2, followed by 25 mg once daily during weeks 3 and 4.^[8]^ In this patient, lamotrigine was initiated directly at 50 mg once daily and was not consistent with this reduced starting and titration schedule. This dosing pattern, the 31-day latency, and the absence of another newly introduced high-risk drug support lamotrigine as the most likely culprit.

Using the algorithm of drug causality for epidermal necrolysis method,^[9]^ the score for lamotrigine was 5 (probable): a 31-day interval from initiation to eruption was compatible (+2); the drug was present in the body on the index day (0); previous exposure was unknown (0); the dechallenge criterion contributed 0; and lamotrigine has a strong association with epidermal necrolysis (+3). The concomitant drugs (valproate, aspirin, and atorvastatin) were also assessed individually. Because each had been initiated more than 56 days before the index day, their intermediate algorithm of drug causality for epidermal necrolysis scores were lower than that for lamotrigine; therefore, no more plausible alternative drug cause was identified (0). Severity-of-illness score for toxic epidermal necrolysis was originally validated using data obtained within the first 24 hours of admission and has also been evaluated serially during the first days of hospitalization.^[10,11]^ In this patient, it was calculated when the SJS/TEN spectrum was first clinically recognized on hospital day 7 rather than at admission; the score of 1 should, therefore, be interpreted cautiously. Skin biopsy was declined, and the diagnosis was based on the clinical evolution, mucosal involvement, positive Nikolsky sign, and progression to approximately 17% epidermal detachment by hospital day 12.

The clinical course demonstrates a stepwise treatment strategy in relapsing SJS/TEN. Initial intravenous immunoglobulin plus methylprednisolone produced transient improvement, whereas subsequent progression prompted corticosteroid escalation and rhTNFR-Fc. The recurrence of erythematous lesions during corticosteroid tapering then prompted TPE as rescue therapy. Further cumulative high-dose corticosteroid exposure was also a concern given his diabetes, hypertension, and previous cerebral infarction. TPE has been used as rescue treatment in refractory SJS/TEN; however, its proposed mechanisms and clinical efficacy remain uncertain.^[2,12]^ Nationwide observational studies have not demonstrated a mortality advantage for early TPE or superiority to intravenous immunoglobulin after ineffective corticosteroid therapy.^[5,13]^ The American Society for Apheresis classifies TPE for refractory TEN as Category III, Grade 2B, supporting individualized rather than routine use.^[14]^ In this patient with SJS/TEN overlap, erythema cleared after 3 TPE sessions while corticosteroid therapy continued; this temporal association does not establish the independent efficacy of TPE. This sequence provides a clinically relevant example of TPE integrated into multidisciplinary rescue management for relapsing disease.

This report is limited by its single-patient design, the absence of histopathologic confirmation, concurrent and sequential systemic therapies, and the 3-month follow-up period. In particular, the use of intravenous immunoglobulin, the concurrent dual-route corticosteroid regimen, rhTNFR-Fc, and TPE prevents attribution of the clinical outcome to any single intervention. These factors should be considered when interpreting the treatment course and its generalizability.

## 4. Conclusion

Direct initiation of lamotrigine at 50 mg once daily during valproate co-therapy exceeded the recommended starting regimen and may have increased the risk of SJS/TEN overlap. This case illustrates the evolution from an initially suspected severe drug eruption to SJS/TEN overlap and describes stepwise multidisciplinary rescue management incorporating culprit-drug withdrawal, supportive care, intravenous immunoglobulin, corticosteroids, rhTNFR-Fc, and TPE. It reinforces careful lamotrigine titration during valproate co-therapy and highlights individualized escalation when the initial response is transient or relapse occurs during treatment reduction.

## Author contributions

**Conceptualization:** Fang Chen, Xiao Li.

**Data curation:** Fang Chen.

**Investigation:** Fang Chen.

**Visualization:** Fang Chen.

**Resources:** Xiao Li.

**Supervision:** Xiao Li.

**Validation:** Xiao Li.

**Writing – review & editing:** Xiao Li.

**Writing – original draft:** Fang Chen.
